# *Helianthus maximiliani* and species fine‐scale spatial pattern affect diversity interactions in reconstructed tallgrass prairies

**DOI:** 10.1002/ece3.5696

**Published:** 2019-10-09

**Authors:** Thomas P. McKenna, Jack McDonnell, Kathryn A. Yurkonis, Caroline Brophy

**Affiliations:** ^1^ Kansas Biological Survey University of Kansas Lawrence KS USA; ^2^ Department of Mathematics and Statistics Maynooth University Maynooth Ireland; ^3^ Department of Biology University of North Dakota Grand Forks ND USA

**Keywords:** Biodiversity‐Ecosystem Function, conspecific aggregation, Diversity‐Interactions modeling, plant–plant interactions, tallgrass prairie

## Abstract

1. Biodiversity and Ecosystem Function analyses aim to explain how individual species and their interactions affect ecosystem function. With this study, we asked in what ways do species interact, are these interactions affected by species planting pattern, and are initial (planted) proportions or previous year (realized) proportions a better reference point for characterizing grassland diversity effects?
2. We addressed these questions with experimental communities compiled from a pool of 16 tallgrass prairie species. We planted all species in monocultures and mixtures that varied in their species richness, evenness, and spatial pattern. We recorded species‐specific biomass production over three growing seasons and fitted Diversity‐Interactions (DI) models to annual plot biomass yields.
3. In the establishment season, all species interacted equally to form the diversity effect. In years 2 and 3, each species contributed a unique additive coefficient to its interaction with every other species to form the diversity effect. These interactions were affected by *Helianthus maximiliani* and the species planting pattern. Models based on species planted proportions better‐fit annual plot yield than models based on species previous contributions to plot biomass.
4. Outcomes suggest that efforts to plant tallgrass prairies to maximize diversity effects should focus on the specific species present and in what arrangement they are planted. Furthermore, for particularly diverse grasslands, the effort of collecting annual species biomass data may not be necessary when quantifying diversity effects with DI models.

## Graphical abstract

We used the Diversity Interaction modeling approach to compare reference point proportion inputs (sown vs. realized proportions) and species arrangement in a multiyear biodiversity experiment. Species interactions are affected by species arrangement, and the extra effort of separating biomass to species (realized proportions) did not provide additional descriptive power.

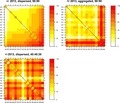

## 1 INTRODUCTION

It is well accepted that plant species and their interactions affect grassland biomass yields (Hector, Bell, Connolly, Finn, & Fox, 2009). Biodiversity‐Ecosystem Function (BEF) analyses aim to quantify these effects and determine to what extent specific species combinations affect yield responses (reviewed in Cardinale et al., 2007; Hector et al., 2009). While simple in concept, this task is mathematically challenging and complicated by increasing interspecific variation (Connolly et al., 2013; Fibich, Rychtecká, & Lepš, 2015).

One approach used to assess diversity effects is Diversity‐Interactions (DI) modeling (Kirwan et al., 2007, 2009). Diversity‐Interactions models quantify species identity effects and diversity effects using a regression framework that combines weighted species monoculture performances with interaction terms to find expected mixture responses (Kirwan et al., 2007, 2009). A general expression of a DI model is.

(1) y=ID+DE+ε

where *y* is the ecosystem function, ID stands for “identity effect” and can be extended to include treatment or block effects, DE stands for “diversity effect”, and *ε* is the error term, typically assumed independent and identically distributed *N*(0,*σ* ^2^).

Derivations of this model include a series of progressively complex DE terms used to characterize alternative species interaction scenarios. The simplest model assumes that species do not interact with one another and that mixture yields are proportional to species monoculture yields (M1: identity model). Additional models allow all species to interact equally, regardless of their species or functional identity (M2: average pairwise model), for each species to contribute uniquely to pairwise interactions regardless of the identity of the other species (M3: additive species‐specific model), and for species to interact differently within and between functional groups (M4: functional group model; details in Methods section). Once these models are fit, model comparison tests are used to determine which interaction scenario best describes observed ecosystem functions.

Diversity‐Interaction models can also test for effects of additional experimental treatments on diversity responses by adding ID and DE interaction terms (Kirwan et al., 2009). This is useful when considering to what extent plant species fine‐scale spatial relationships affect plot‐scale diversity effects. Plant species interact on finite‐scales, and if their interaction distances are small enough, their spatial relationships can presumably affect the interactions they experience and respond to (Houseman, 2014; Lamošová, Doležal, Lanta, & Lepš, 2010; Murrell, 2010; Porensky, Porensky, Vaughn, & Young, 2012; Seahra, Yurkonis, & Newman, 2019; Stoll & Prati, 2001; Yurkonis & McKenna, 2014). In spatially manipulated tallgrass prairies, increasing species interspecific interactions increased biomass and favored clonal forbs (McKenna & Yurkonis, 2016), an effect that was replicated by seeding species in smaller conspecific patches (Seahra, Yurkonis, & Newman, 2016). However, manipulating species pattern had a neutral effect on plot‐scale species interactions (quantified by the Additive Partitioning method; Loreau & Hector, 2001) in these studies (McKenna & Yurkonis, 2016; Seahra et al., 2016). In both cases, species responses to changes in their interaction neighborhoods were likely so species specific that positive and negative pattern effects combined to create a neutral overall diversity effect. Because DI models can expressly quantify species contributions to DE in response to treatments, they provide an avenue to more specifically elucidate how species interaction neighborhoods affect tallgrass prairie diversity.

As with other BEF modeling approaches, DI models require users to a priori determine how each species is expected to proportionally contribute to community effects. Expected species proportions can be set based on the number of individuals (e.g., sown or planted individual proportions) or on the relative size (proportional biomass) of each species in mixture. If species interactions are proportional to their size, setting species proportions based on their relative biomass (e.g., by replacing individual based proportions with first or subsequent year proportional biomass) may improve BEF model fit (Finn et al., 2013; Grace, Keough, & Guntenspergen, 1992; Kirwan et al., 2009). However, this would be ineffective if the outcome of species interactions were unrelated to their aboveground biomass. This could occur if wide swings in species‐specific biomass production occur from year to year to affect the rank‐order of species among growing seasons (Brophy, Finn, et al., 2017; Finn et al., 2013). In this case, diversity effects may be best explained by simply setting species proportions based on their relative number of individuals. While Finn et al. (2013) found that adjusting for species previous year proportional biomass improved DI model fit, using the “planted proportions of individuals” approach may provide a better model fit for communities with large or variable species pools.

In this study, we tested effects of species interactions on plant biomass production by applying DI models to the first three years of biomass data collected from a grassland biodiversity experiment. We address the following questions:

1. Which diversity effect framework (M1–M4) best describes plot biomass?
2. Are diversity effects affected by species planting pattern?
3. Are interactions that affect plot biomass better described based on species individual (planted) proportions or their previous proportional biomass?

## 2 METHODS

### 2.1 Experimental design

**Figure 1:**
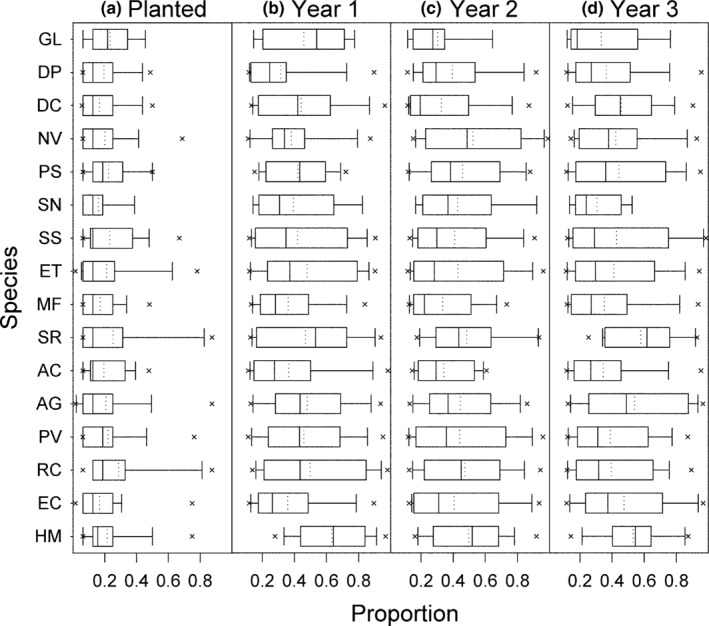
Box plots of species planted and yearly proportions at harvest in mixture plots only across all treatments in the first three years of the SPaCE experiment. Species are labeled with the first letter of their genus and specific epithet (Table 1) and are ordered by their average year 1 monoculture yields. Means are indicated with dotted lines

The Species Pattern and Community Ecology (SPaCE) experiment (North Dakota, USA) consists of 1 m × 1 m field plots planted with greenhouse grown seedlings in June 2012. The field had been in row crop production for the previous 15 years and was planted with spring wheat in the previous growing season. Plots varied in species richness (2, 4, 8 species and monocultures), evenness (low, intermediate, high), and pattern (dispersed and aggregated). Plots were spaced 2 m apart with mown aisles and arranged in a randomized complete block design with five blocks. At planting, we divided each plot into an 8 × 8 grid of 64 planting cells and planted a single seedling in each cell. For the pattern treatment, we either randomly assigned individuals to each of the 64 planting cells (“dispersed” treatment) or we randomly assigned individuals of each species to a 2 × 2 set of four cells (“aggregated” treatment). With this design, we increased conspecific interactions among nearest neighbors in aggregated plots relative to dispersed plots. For the evenness treatment, we altered the number of individuals planted from each species within each richness level. In two‐species plots, the ratio of individuals of each species was either 8:56 (low), 16:48 (intermediate), or 32:32 (high). In four species plots, the ratio of individuals was either 4:4:28:28, 8:8:24:24, or 16:16:16:16. In eight species plots, the ratio of individuals was either 4:4:4:4:4:8:16:20, 4:4:4:4:12:12:12:12, or 8:8:8:8:8:8:8:8. Species were selected from a pool of 16 common tallgrass prairie species (Table 1) and randomly assigned to plots with the following functional group constraints: two‐species plots contained a grass (warm or cool season) and a legume or a forb, four species plots contained one species from each functional group, and eight species plots contained two species from each functional group. We additionally randomly assigned species to abundance levels within each plot, which resulted in some variation across species in their average planted proportions across all treatments (range 16%–28%; Figure 1a).

**Table 1:** The SPaCE experiment species pool consisted of 16 common tallgrass prairie species selected from four functional groups (warm‐season grasses, cool‐season grasses, forbs, and legumes)

Sp. No.	Species	Abbr.	Common name	Functional group
1	*Andropogon gerardii*	AG	Big bluestem	Warm‐season grass
2	*Schizachyrium scoparium*	SS	Little bluestem	Warm‐season grass
3	*Sorghastrum nutans*	SN	INDIAN grass	Warm‐season grass
4	*Panicum virgatum*	PV	Switchgrass	Warm‐season grass
5	*Elymus Canadensis*	EC	Canada wild rye	Cool‐season grass
6	*Elymus trachycaulus*	ET	Slender wheatgrass	Cool‐season grass
7	*Pascopyrum smithii*	PS	Western wheatgrass	Cool‐season grass
8	*Nassella viridula*	NV	Green needle grass	Cool‐season grass
9	*Monarda fistulosa*	MF	Wild bergamot	Forb
10	*Solidago rigida*	SR	Stiff goldenrod	Forb
11	*Helianthus maximiliani*	HM	Maximilian sunflower	Forb
12	*Ratibida columnifera*	RC	Yellow coneflower	Forb
13	*Desmodium canadense*	DC	Showy tick trefoil	Legume
14	*Astragalus canadensis*	AC	Canada milkvetch	Legume
15	*Dalea purpurea*	DP	Purple prairie clover	Legume
16	*Glycyrrhiza lepidota*	GL	American licorice	Legume

We weeded the plots monthly during the growing season to remove volunteers from the local propagule pool and any nonassigned species from the study species pool. At the end of each of the three growing seasons (September 2012, 2013, and 2014), aboveground biomass was cut to 5 cm above the soil surface, sorted to species, dried to a constant mass (60°C), and weighed. Further experimental details are provided in McKenna and Yurkonis (2016).

We used total and species‐specific aboveground biomass data from 170 plots of the SPaCE experiment for this analysis (3 levels richness × 3 levels evenness × 2 levels species spatial pattern = 18 mixtures + 16 monocultures = 34 plots × 5 blocks = 170 plots). For each plot, we calculated the planted proportion of each species (planted proportion = # individuals for species *i*/64 subplots) and the proportional biomass of each species in each year (realized proportion = harvest biomass of species *i*/total plot biomass; Figure 1b–d). We used species planted proportions as predictors in our Diversity‐Interactions (DI) modeling analysis of year 1 total plot biomass. We used species planted proportions and separately used species realized proportions in our DI modeling analysis of year 2 and year 3 total plot biomass.

### 2.2 Application of Diversity‐Interactions (DI) models to SPaCE data

We considered a series of four hierarchical DI models to describe yearly plot biomass and test alternative hypotheses about species interactions in the plots. These models were related to a realization of Equation (1), the “full pairwise interaction” model wherein.

(2) $$y=\sum_{i=1}^{S}\beta_{i}P_{i}+\alpha_{b}+\sum_{\begin{array}{c} i,j=1\\ i<j \end{array}}^{S}\delta_{\mathrm{ij}}P_{i}P_{j}+\varepsilon$$

where *y* is total plot biomass, *S* = 16 is the total number of species in the pool (Table 1), *P_i_* is the reference proportion of species *i* (either planted or realized in the preceding year), *β_i_* is the expected monoculture yield of species *i*, *α_b_* is the effect of block *b*, where *b* = 1,…,5, *δ_ij_* is the potential for two species to interact, *δ_ij_P_i_P_j_* is the contribution to biomass resulting from the interaction of species *i* and *j* and *ε* is assumed i.i.d. *N*(0,*σ* ^2^). Since there are a large number of possible pairwise interactions for this 16‐species system $\left(120=\left(\begin{array}{c} 16\\ 2 \end{array}\right)\right)$, we considered four DI models that imposed some constraints among the *δ_ij_* (DE) coefficients (Kirwan et al., 2009).

Model 1 (M1): The “identity” model assumed that species do not interact with one another, *that is δ_ij_* = 0 for all *i*, *j*. In monoculture, the expected performance of species *i* is *β_i_*, adjusted for block. In mixture, the expected plot yield is a weighted average of the species expected monoculture performances, adjusted for block, and it is assumed that there are no interaction effects. The equation is:
  (M1) $$y=\sum_{i=1}^{S}\beta_{i}P_{i}+\alpha_{b}+\varepsilon$$
Model 2 (M2): The “average pairwise interaction” model assumed all pairs of species interacted with one another in equal strength, that is*. δ_ij_* = *δ* for all *i*, *j*. The equation is:
  (M2) $$y=\sum_{i=1}^{S}\beta_{i}P_{i}+\alpha_{b}+\delta \sum_{\begin{array}{c} i,j=1\\ i<j \end{array}}^{S}P_{i}P_{j}+\varepsilon$$
Model 3 (M3): The “additive species‐specific interactions” model assumed that each species contributed a unique and constant (additive) coefficient to its interaction with every other species, regardless of the identity of the other species in the interaction. The expected interaction for any pair of species is the sum of their two unique coefficients, *that is δ_ij_* = *λ_i_* + *λ_j_* for all *i*, *j*. The equation is:
  (M3) $$y=\sum_{i=1}^{S}\beta_{i}P_{i}+\alpha_{b}+\sum_{\begin{array}{c} i,j=1\\ i<j \end{array}}^{S}\left(\lambda_{i}+\lambda_{j}\right)P_{i}P_{j}+\varepsilon$$
Model 4 (M4): The “functional group” model assumed that pairs of species from the same functional group *k* (*k* = 1,…,4; cool‐season grass, warm‐season grass, forb, and legume; Table 1) interacted in the same way, that is *δ_ij_* = *ω_kk_* for all *i* ≠ *j* from the *k*th functional group, and that pairs of species from different functional groups *k* and *l* interacted in the same way, that is *δ_ij_* = *ω_kl_* for all *i* from the *k*th functional group and all *j* from the *l*th functional group. Thus, the functional group model included ten interaction parameters, four “within functional group” interactions: *ω*_11_, *ω*_22_, *ω*_33_, *ω*_44_, and six “between functional group” interactions: *ω*_12_, *ω*_13_, *ω*_14_, *ω*_23_, *ω*_24_, *ω*_34_. The equation is:
  (M4) $$y=\sum_{i=1}^{S}\beta_{i}P_{i}+\alpha_{b}+\sum_{k=1}^{T}\omega_{\mathrm{kk}}\sum_{\begin{array}{c} i,j\in FG_{k}\\ i<j \end{array}}P_{i}P_{j}+\sum_{\begin{array}{c} k,l=1\\ k<l \end{array}}^{T}\omega_{\mathrm{kl}}\sum_{i\in FG_{k}}\sum_{j\in FG_{l}}P_{i}P_{j}+\varepsilon$$

Where *T* = 4 and FG_1_ = {1,2,3,4}, FG_2_ = {5,6,7,8}, FG_3_ = {9,10,11,12}, FG_4_ = {13,14,15,16} and the species names are given in Table 1. Models ((M1), (M2), (M3), (M4)) inherently account for the SPaCE experiment richness and evenness treatments by including the species present and their expected plot‐scale proportions when predicting plot biomass yields. To account for the species pattern treatment, we expanded these models to test for interactions of the pattern treatment with DE terms in mixture plots. For example, the expanded version of model 2 (M2) is:

(3) $$y=\sum_{i=1}^{S}\beta_{i}P_{i}+\alpha_{b}+\delta_{1}\sum_{\begin{array}{c} i,j=1\\ i<j \end{array}}^{S}P_{i}P_{j}+\delta_{2}\left(\sum_{\begin{array}{c} i,j=1\\ i<j \end{array}}^{S}P_{i}P_{j}\right)\times \text{Sp}+\varepsilon$$

where spatial pattern (Sp) is coded 1 for aggregated plots, 0 for dispersed plots, and 0 for monocultures. The term *δ_1_* is the interaction between any pair of species in dispersed plots. The term *δ_2_* allows for a change in the interaction between any pair of species in aggregated plots.

We expanded the model further to test for interactions of the tall‐statured, rhizomatous forb, *Helianthus maximiliani* (HM, maximilian sunflower, species # 11 in Table 1) with the DE terms. For example, the expanded version of Equation (3) is:

(4) $$y=\sum_{i=1}^{S}\beta_{i}P_{i}+\alpha_{b}+\delta_{1}\sum_{\begin{array}{c} i,j=1\\ i<j \end{array}}^{S}P_{i}P_{j}+\delta_{2}\left(\sum_{\begin{array}{c} i,j=1\\ i<j \end{array}}^{S}P_{i}P_{j}\right)\times \text{Sp}+\delta_{3}\left(\sum_{\begin{array}{c} i,j=1\\ i<j \end{array}}^{S}P_{i}P_{j}\right)\times P_{11}+\varepsilon$$

**Figure 2:**
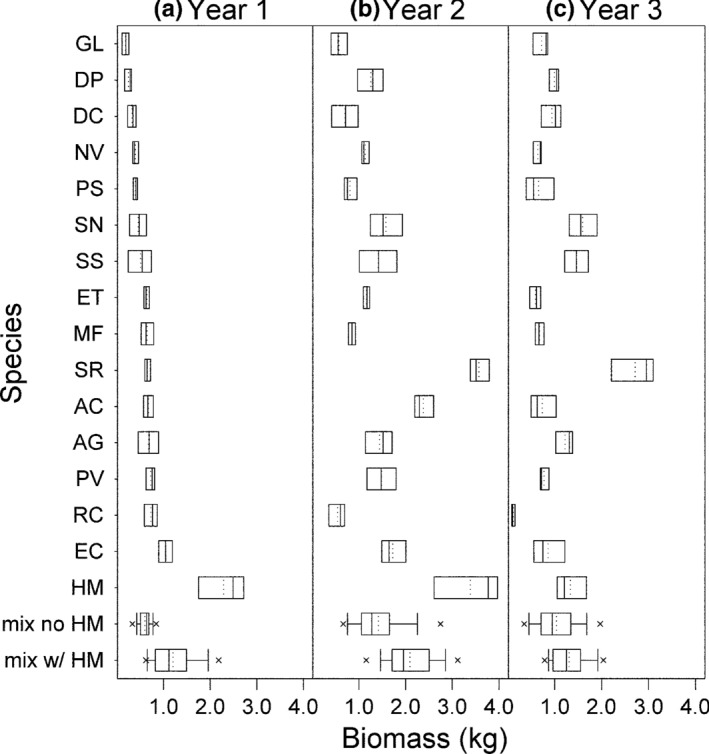
Box plots of species monoculture (*n* = 5) and mixture yields over the first three years of the SPaCE experiment. Mixtures were separated by those with and without *Helianthus maximiliani* (HM). Species are ordered by their average monoculture yields in year 1. Means are indicated by dashed lines

The term *δ_3_* in Equation (4) allows for a change in the species pairwise interactions between mixtures with and without *H. maximiliani* by scaling *δ_3_* by its reference (planted or realized) proportion (*P*_11_; *P*_11_ = 0 for mixtures without species 11). This statistical three‐factor interaction allows for a nonsymmetric species interaction effect as the proportion of *H. maximiliani* changes. We treated *H. maximiliani* separately because of lack‐of‐fit in models that did not include these additional interactions. Specifically, we identified patterns in residuals related to the proportion of *H. maximiliani*. This was not surprising, since *H. maximiliani* was considerably more productive than the other species in monoculture and *H. maximiliani* mixtures were more productive and more variable than non‐ *H. maximiliani* mixtures (Figure 2).

We fitted Models ((M1), (M2), (M3), (M4)) separately for each year following a hierarchical process (details in Appendix S1). We used species planted proportions as the predictors for year 1 and separately used species planted proportions and realized proportions as predictors for years 2 and 3. Raw data visualizations (Figure 2) and initial model diagnostic tests showed that the error variances were not constant for all models. More flexible error structures that allowed the variance to change depending on plot characteristics (such as whether or not the plot was a monoculture, or whether or not *H. maximiliani* was included in a mixture) were tested and used where required (Brophy, Dooley, et al., 2017; Connolly et al., 2018); details in Appendix S1. We used *F*‐tests or likelihood ratio tests (as appropriate) for model comparisons throughout the model fitting process (details in Appendix S1). After we identified the best fixed effects model, we determined if any additional variation could be accounted for by including all pairwise interactions as random effects, each constrained to have the same variance (Brophy, Dooley, et al., 2017). If random effects were needed in the model, this approach accounted for the remaining uncertainty in a parsimonious way. We carried out all analyses in SAS version 9.3 or SAS University Edition (SAS Institute Inc., Cary, NC, USA).

## 3 RESULTS

### 3.1 Year 1

In the first growing season, M2, the average pairwise model, modified so that the DE term interacted with the planted proportion of *H. maximiliani* was the best model (Table 2; full model specification in Appendix S1). With this model, the diversity effect increased as *H. maximiliani* increased. For example, the estimated DE for a 4‐species community without *H. maximiliani* and with 25% of each species was 35.1 ± 15.82. This increased sevenfold in the presence of *H. maximiliani* such that when one of the four species was *H. maximiliani*, the estimated DE was 255.19 ± 59.9. This positive DE means that there is a positive benefit to mixing species beyond what they contribute to plot biomass based on their monoculture performance. In this case, diversity effects were not determined by the species present, their functional identity, or their spatial pattern beyond the effects of *H. maximiliani*.

**Table 2:** Summary of the best‐fit models in each of the three years of the SPaCE experiment. Model selection followed a hierarchical process (outlined in methods, Appendix S1, and in Table S1.1). We tested the best model under each year‐proportion scenario for additional interactions between the diversity effect terms (DE) and the study species spatial pattern treatment (Sp) and the proportion of *Helianthus maximiliani* (P_11_). Best‐fit planted and realized models were compared with AIC in years two and three. Conditions of the final best model in each year are indicated in bold text. The number of fixed parameters is shown for each model as: # identity (ID) terms + # block terms + # diversity effect (DE) terms

Year	Proportion	Best model	Additional interactions	No. Parameters	ΔAIC
1	Planted	**M2: Average pairwise**	(DE) * P_11_	16 + 4 + 2	
2	Planted	**M3: Additive species**	(DE) * P_11_ + (DE) * Sp	16 + 4 + 48	
	Realized	M3: Additive species	(DE) * P_11_ + (DE) * Sp	16 + 4 + 48	16.5
3	Planted	**M3: Additive species**	(DE) * P_11_ + (DE) * Sp + Random Pairwise	16 + 4 + 48	
	Realized	M2: Average pairwise	–	16 + 4 + 1	16.5

### 3.2 Year 2

By the end of the second growing season, the identity of the species present and their spatial pattern affected plot biomass. In year 2, M3, the additive species‐specific model, based on species planted proportions and with DE terms interacting with both the proportion of *H. maximiliani* and the spatial pattern treatment was the best model (Table 2, Appendix S1). This model assumed that each species contributed a unique and constant coefficient to its interaction with every other species and that this species‐specific contribution changed in the presence of *H. maximiliani* and with the spatial pattern treatment. We found no evidence of species‐specific contributions to biomass being related to their functional identities.

**Figure 3:**
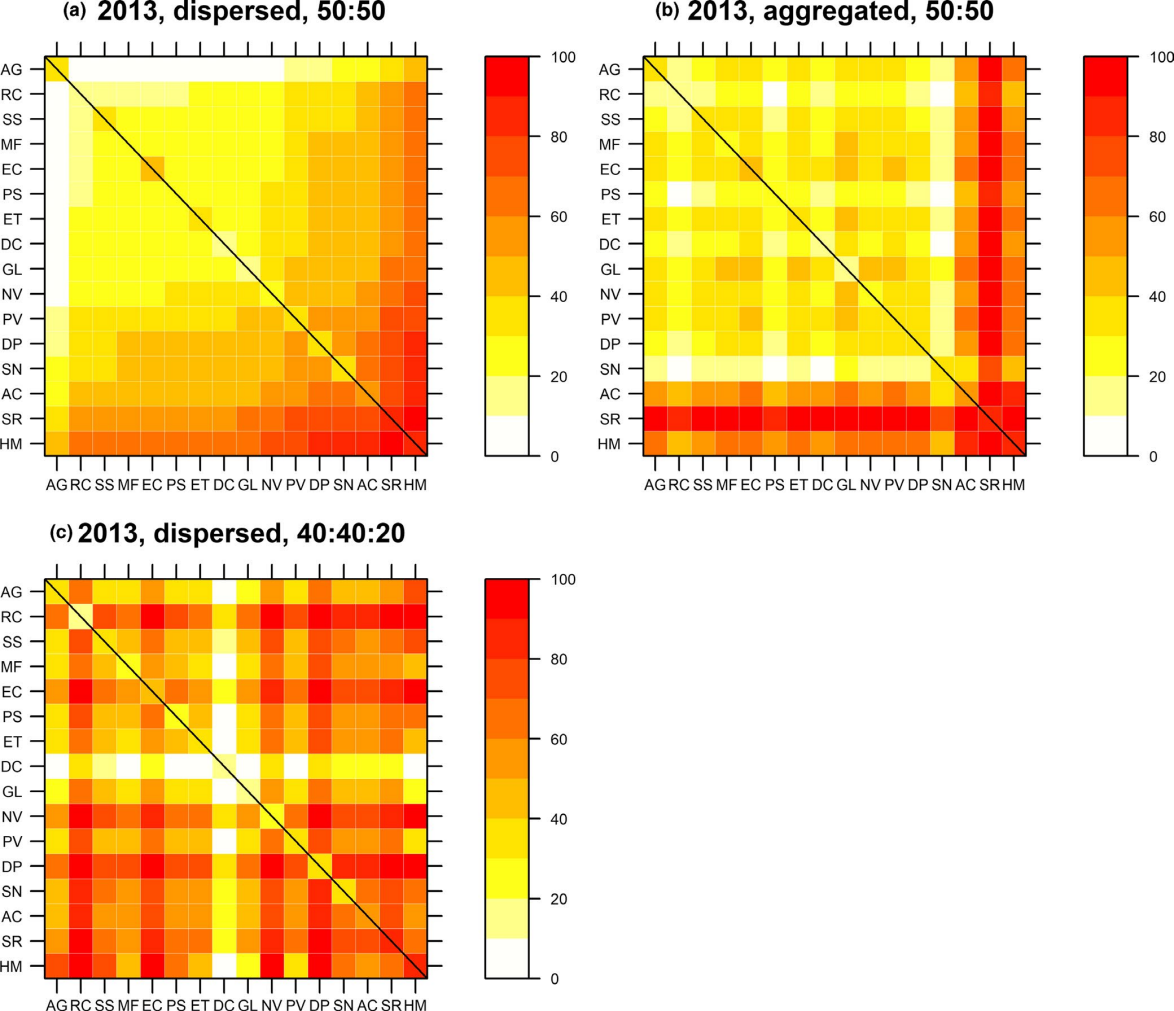
Predicted monoculture (diagonal squares highlighted by black line) and mixture (off‐diagonal) biomass from the best‐fit Diversity‐Interactions model of plot biomass in year 2 (2013) of the SPaCE experiment. Values are expressed as a percentage of the maximum observed plot biomass across the first three years of the experiment (4,080.87 g in a *Helianthus maximiliani* monoculture plot in year 2). The diagonals are the same in each panel. The off‐diagonals in panels (a) and (b) show scaled predicted biomass for 50:50 mixtures of the two species in (a) dispersed and (b) aggregated species spatial patterns. Panel (c) shows predicted biomass for 40:40 mixtures of the two species plus 20% of *H. maximiliani* (HM) in the dispersed species spatial pattern. Species are labeled by abbreviations listed in Table 1 and ordered by increasing predicted value on the *x*‐axis in panel (a)

We used heat maps to visualize predicted total plot biomass from the best‐fit model fitted to the full dataset and without any out of sample verification (Figure 3). For simplicity, we show total predicted biomass (ID and DE combined) for two‐species (50:50) plots and three‐species plots with 20% *H. maximiliani* (40:40:20). Two‐species (50:50) plots were predicted to be most productive when containing the larger statured species, *H. maximiliani*, *Solidago rigida*, and *Astragalus Canadensis* (Figure 3a). *Andropogon gerardii* and *S. rigida* were predicted to benefit the most when planted in an aggregated species pattern compared with the dispersed species pattern. In contrast, *Sorghastrum nutans* was predicted to suffer the most under the aggregated species pattern treatment (Figure 3a vs. 3b). When 20% of *H. maximiliani* was introduced into any two‐species mixture, there was an automatic predicted benefit since *H. maximiliani* was one of the highest yielding species in year 2. This effect was picked up via the ID effect in the best‐fit DI model and can be seen by the generally “redder” coloring of Figure 3a versus 3c. Including an additional 20% of *H. maximiliani* was also predicted to improve the ability of *A. gerardii*, *Ratibida columnifera* and *Elymus Canadensis* to interact with other species and reduce the ability of *Desmodium canadense* to interact with other species (Figure 3a vs. 3c).

### 3.3 Year 3

By the end of the third growing season, the identity of the species present and their spatial pattern continued to affect plot biomass. In year 3, M3, the additive species‐specific model, based on species planted proportions with DE terms interacting with the proportion of *H. maximiliani* and the spatial pattern treatment was again the best model (Table 2, Appendix S1). In this case, including random pairwise interaction terms improved model fit (Table 1). Again, we had no evidence of the species‐specific contributions to biomass being related to their functional identities.

**Figure 4:**
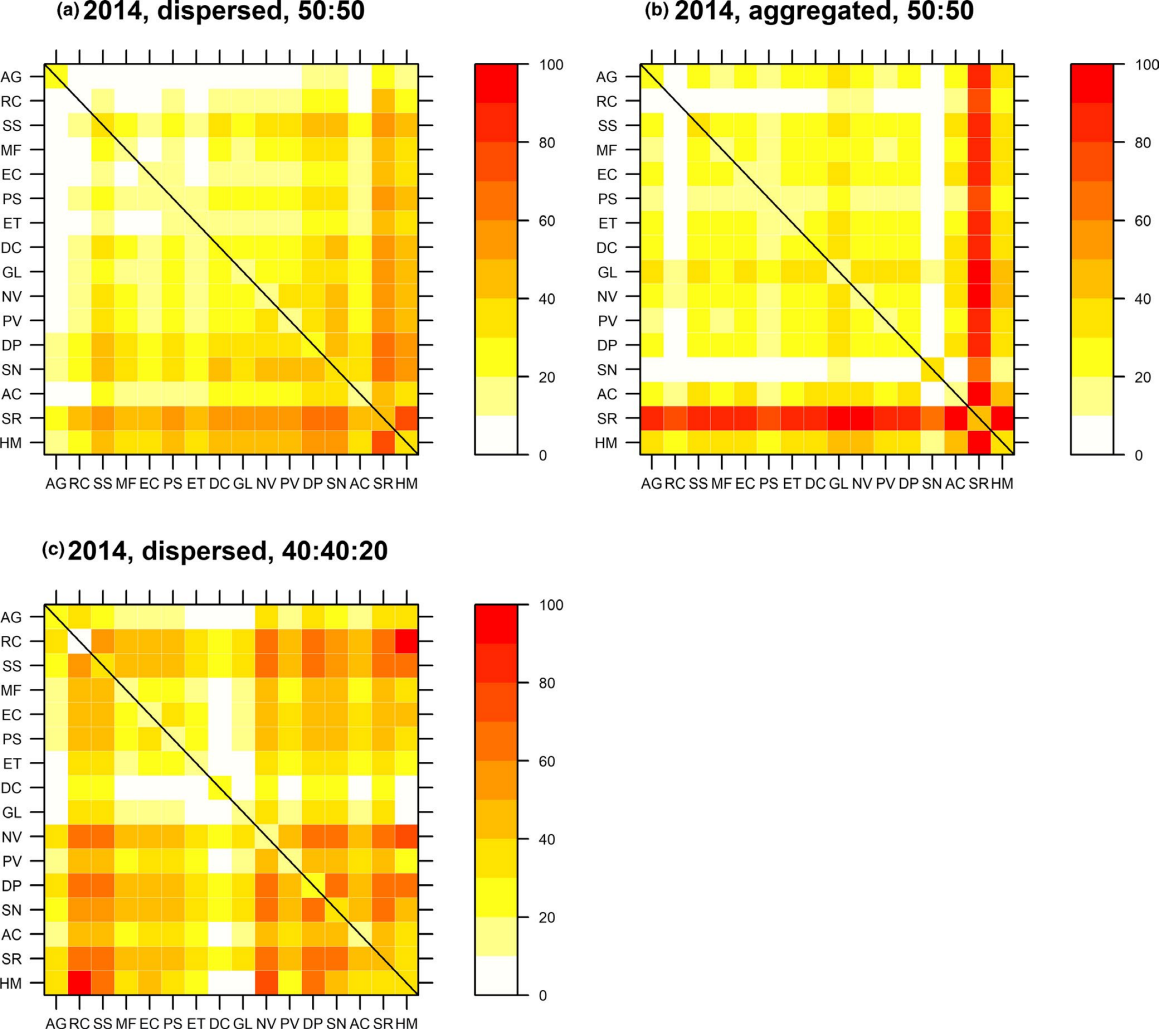
Predicted monoculture (diagonal squares highlighted by black line) and mixture (off‐diagonal) biomass from the best‐fit Diversity‐Interactions model of plot biomass in year 3 (2014) of the SPaCE experiment. Values are expressed as a percentage of the maximum observed biomass (4,080.87 g in a *Helianthus maximiliani* monoculture plot in year 2). The diagonals are the same in each panel. The off‐diagonals in panels (a) and (b) show scaled predicted biomass for 50:50 mixtures of the two species in (a) dispersed and (b) aggregated species spatial patterns. Panel (c) shows predicted biomass for 40:40 mixtures of the two species plus 20% of *H. maximiliani* (HM) in the dispersed species spatial pattern. Species are labeled by abbreviations listed in Table 1 and ordered by increasing predicted value on the *x*‐axis in Figure 3a

Although the best‐fit model was similar between years 2 and 3, predicted biomass yields from the year 3 model were generally lower relative to the year 2 model (Figure 3 vs. 4). In year 3, *A. gerardii*, *S. rigida* (as in year 2) and *A. canadensis* were predicted to benefit the most from increased intraspecific interactions under the aggregated species pattern treatment. *Sorghastrum nutans* was again predicted to suffer the most under the aggregated spatial pattern treatment (Figure 4a vs. 4b). Including an additional 20% of *H. maximiliani* was again predicted to improve the ability of *R. columnifera* to interact with other species and reduce the ability of *D. canadense* to interact with other species (Figure 4a vs. 4c).

## 4 DISCUSSION

We used Diversity‐Interactions models to quantify diversity effects in each of the first three years of a grassland biodiversity experiment. Species interactions that contributed to diversity effects developed over time and differed among species. In the first growing season, aside from the most productive forb, *H. maximiliani*, species interacted equally to positively affect plot biomass. In the second growing season, each species contributed a unique constant to interactions that affected plot biomass. These species interaction constants were affected by *H. maximiliani* and the species planting pattern. This continued into the third growing season. In all three years, plot biomass was best described when species expected proportions were set based on their individual (planted) proportions. It appears that species interactions that form grassland diversity effects are affected by the fine‐scale neighborships among species and can be modified in the presence of even a single, high‐performing species. Our results suggest that managers need to consider species‐specific responses, as opposed to species functional identities, when planning for diversity effects in species‐rich reconstructed grasslands.

### 4.1 Helianthus maximiliani effects

As in other studies (Dickson & Busby, 2009; Kordbacheh, Jarchow, English, & Liebman, 2019; Nemec, Allen, Helzer, & Wedin, 2013; Seahra et al., 2019), a productive Heliantheae forb initially dominated our experimental tallgrass prairies reconstructed on a former agricultural field. To address this statistically, we allowed diversity effects in our DI models to differ in *H. maximiliani* plots and treated these plots with a different variance structure. This additional statistical treatment and interpretation of *H. maximiliani* effects was biologically warranted. In the context of the SPaCE experiment, we previously reported that this species contributed to strong selection effects in the first and second growing seasons (McKenna & Yurkonis, 2016). In general, the early dominance of *H. maximiliani* in reconstructed grasslands has been correlatively attributed to strong competitive and potentially allelopathic effects on co‐occurring species (Dickson & Busby, 2009; Kordbacheh et al., 2019; Macías, Torres, JoséM, Molinllo, & Castellano, 1996; Nemec et al., 2013). However, evidence of a direct effect on other species is lacking in the literature.

This improved, yet temporary, productivity likely comes from how Heliantheae forbs capture belowground resources within former agricultural fields. The related crop species *Helianthus annuus*, domesticated from North American *Helianthus* species, is deep rooted (reported up to 3 m), with a faster advance rate and better water use efficiency than comparable warm‐season, cool‐season, and legume crops (Canadell et al., 1996; Krupinsky, Tanaka, Merrill, Liebig, & Hanson, 2006; Stone, Goodrum, Jaafar, & Khan, 2001; Stone, Goodrum, Schlegel, Jaafar, & Khan, 2002). Anecdotally, these annual sunflowers are known to forage for nitrogen leached beyond the typical crop root zone and are planted to recover “lost” nitrogen in agricultural systems. What limited research exists supports this assertion (Canadell et al., 1996; Corbeels, Hofman, & Cleemput, 1998; López‐Bellido, López‐Bellido, Castillo, & López‐Bellido, 2003; Moore & Peterson, 2007). Interestingly, in a North Dakota, USA row crop species rotation study, annual sunflower was the only nonlegume whose residue enhanced subsequent crops (Krupinsky et al., 2006), an effect potentially related to higher leaf nutrient content (Fässler et al., 2010). It is possible that the increased *H. maximiliani* productivity in our study resulted from this greater, more rapid nitrogen and soil water acquisition from deeper in the soil profile. Wang's (2008) work in similar North Dakota, USA grasslands also appears to support this assertion in that *H. maximiliani* had high root decomposition and mixtures with *H. maximiliani* had greater root decomposition than those without. Although we did not sample soil or plant tissue nutrients, growing season soil moisture was approximately 4% higher in *H. maximiliani* mixtures relative to non‐*H. maximiliani* mixtures in all three years (2012 *F*_1,88_ = 4.29, *p* < .05; 2013 *F*_1,88_ = 3.40, *p* = .07; 2014 *F*_1,88_ = 3.62, *p* = .06; methods described in McKenna & Yurkonis, 2016).

Given its potential contribution to the water and nutrient balance of an entire plot, it makes sense that *H. maximiliani* altered interactions among the remaining species. Our results imply that its dominance early in the reconstruction process should be interpreted with caution in that high *H. maximiliani* biomass production does not necessarily result in detrimental effects to other species. Future studies need to consider its role in accessing belowground resources in the restoration context and its use in regulating nutrient dynamics in grassland agroecosystems.

### 4.2 Spatial pattern effects

As with others (Lamošová et al., 2010; Seahra et al., 2016; Stoll & Prati, 2001), we found that aggregating plant species at submeter scales alters grassland species interactions, particularly among subordinate, less productive species. When using ANOVA type approaches to analyze this data, we previously identified a weak overall effect of species pattern on biomass production and no diversity effect as determined by the Additive Partitioning BEF analysis method (McKenna & Yurkonis, 2016). While this net effect is useful from a total biomass production perspective, the approach used here demonstrates the value of DI models to highlight species‐specific spatial pattern effects on diversity effects. With this analysis, *A. gerardii*, *S. rigida,* and *S. nutans* were predicted to be most consistently affected by species planting pattern, but in varying ways. These varied effects may be due to differences in how species interact with local resources and their soil biota under different planting arrangements (McKenna, Darby, & Yurkonis, 2019; Schnitzer et al., 2011; Temperton, Mwangi, Scherer‐Lorenzen, Schmid, & Buchmann, 2007; Van der Putten et al., 2013). While we were unable to determine the mechanisms behind these predicted species‐specific responses to species patterns, the DI modeling framework was useful for identifying which species should be further considered when applying these findings in a restoration context.

### 4.3 Planted versus realized proportions

From our study, it appears that using species planted proportions is sufficient for characterizing diversity effects in the DI framework for reconstructed grassland communities. It is well documented that diversity effects are sensitive to variation in species yields (Fargione et al., 2007; Polley, Wilsey, & Derner, 2003) that arise from biotic (i.e., plant‐soil feedbacks) and abiotic sources (i.e., changes in resource availability, variation in plant phenology at harvest). These factors can change the rank‐order among species and their proportional contributions to mixtures among years without affecting species interactions that affect community biomass (Loreau & Hector, 2001). For example, in our study, *S. rigidia* did not bolt from the basal rosette stage prior to harvest in year 1, but did so prior to harvest in years 2 and 3. This affected the proportion of *S. rigidia* in mixture among years, but likely bore little effect on how *S. rigidia* interacted with others within each growing season. *Helianthus maximiliani* and *A. canadensis* had similar swings in their biomass that likely did not affect how they interacted with other species over time. Although species biomass proportions have been used to improve DI models in communities with less variation among species and greater temporal stability (Fibich et al., 2015; Finn et al., 2013), it appears that this is ineffective for predicting peak biomass in communities with high variation among species and over time. This is especially important given that there is such a high labor cost to obtaining annual species‐specific yield data, which were ultimately less informative.

### 4.4 Tallgrass prairie diversity effects

In applying DI models to species‐rich tallgrass prairies, we expand upon a developing literature on species‐specific grassland diversity effects, and affirm that species‐specific interactions drive diversity effects in species‐rich grasslands. Diversity‐Interactions models were first applied to assess biomass production in a pasture experiment seeded with two grasses and two legumes. Given the small and relatively similar species pool, it was not surprising that the average pairwise model (M2) was consistently the best‐fit model for this agroecosystem (Finn et al., 2013; Kirwan et al., 2007). In applying DI models to assess biomass production within a nine‐species grassland experiment, Brophy, Dooley, et al. (2017) found that more complex species‐specific interactions (e.g., functional group with random pairwise interaction) contributed to grassland diversity effects. We affirm this finding in that M3 consistently best explained plot biomass with an even larger species pool. These results indicate that while diversity effects exist, potential species contributions must be evaluated on a species‐by‐species basis when planning for emergent grassland diversity effects in the grassland reconstruction process.

### 4.5 Management implications

The findings have implications for grassland reconstruction efforts. First, it is clear that multiple species are needed to maximize diversity effects in reconstructed tallgrass prairies. While many studies have demonstrated this effect (Cardinale et al., 2007; Isbell, Polley, & Wilsey, 2009; Seahra et al., 2016), our study is unique in that we quantified specific species contributions to diversity effects. Specifically, we found that including *H. maximiliani* can enhance diversity effects, albeit it can appear that it outcompetes others based on its initially high biomass production (Dickson & Busby, 2009; Kordbacheh et al., 2019; Nemec et al., 2013). Second, because species broad functional identities (cool‐season, warm‐season, forb, legume) were not informative, managers may best approach planning for tallgrass prairie diversity effects by keeping individual species characteristics, as opposed to their broad functional identities, in mind. Finally, it is necessary for managers to take a species‐specific approach to planning planting activities in order to take advantage of the benefits of aggregating select species (e.g., *S. rigida*, *A. gerardii*).

## CONFLICT OF INTEREST

None declared.

## AUTHOR'S CONTRIBUTIONS

KAY and TPM designed and established the experiment. TPM coordinated data collection. CB and JM designed the data analysis methods. CB and JM analyzed the data with contributions from KAY and TPM. TPM, KAY, and CB wrote the manuscript with assistance from JM.

## Data Availability

Data used for the analysis in this manuscript are available in the Dryad Repository: https://doi.org/10.5061/dryad.r2h6h33
