# Supplementary material for: Helianthus maximiliani and species fine‐scale spatial pattern affect diversity interactions in reconstructed tallgrass prairies
Source: Ecol Evol. 2019 Oct 9;9(21):12171–81. doi: 10.1002/ece3.5696 (PMC6854329; doi:10.1002/ece3.5696)
Supplement: Supplementary file 1 [file ECE3-9-12171-s001.docx]

**Appendix S1**

In this appendix, we describe the model fitting process in further detail and provide the full specification of the DI model selected in each year-proportion case (year 1 planted, year 2 planted, year 2 realized, year 3 planted, and year 3 realized). In each case, *y* is the plot aboveground biomass (g) yield, S is the total number of species and is equal to 16, P*_i_* is the proportion (planted or realized) of species *i*.

*Additional Methods*

We followed a six-step model fitting procedure to select the model that best described plot biomass in each case (year 1 planted, year 2 planted, year 2 realized, year 3 planted, and year 3 realized). The steps were:

1. We fitted the four DI models (M1-M4 in the main text) to plot biomass production in each case. These models were fit using least squares and assumed constant variance across all plots. We selected the best models using F-tests and visually inspected their residual plots to determine if model assumptions were met. In all cases, constant residual variance assumptions were not met for the best-fit models identified at this step (Fig. S1.1, constant variance panels; where a ‘fanning’ pattern is observed in the residuals versus predicted plots). Additional residual plots (e.g. residual versus the proportion of individual species; not shown) showed patterns among residuals related to the variation in monoculture and mixture yields and whether or not *H. maximiliani* (HM) was included in a mixture.
2. We tested five alternate more flexible error structures for each best-fit model using REML (restricted maximum likelihood) estimation. We allowed the error variance to change under each of the following scenarios:

- Monocultures, mixtures (2 parameters)
- *H. maximiliani* monocultures, other monocultures, mixtures (3 parameters)
- *H. maximiliani* monocultures, other monocultures, mixtures with *H. maximiliani*, other mixtures (4 parameters)
- Each of the 16 monocultures, mixtures (17 parameters)
- Each of the 16 monocultures, mixtures with *H. maximiliani*, other mixtures (18 parameters)

1. We re-fit the four DI models (M1-M4 in the main text) using ML (maximum likelihood) estimation using the best error structure identified in step 2 and used likelihood ratio tests to select the best fit model.
2. Building on the best-fit model identified in step 3, we tested if additional interactions with the diversity effect coefficients were needed. This included testing for an interaction between the diversity effects coefficients with each of the species spatial pattern treatment and the influential species *H. maximiliani*.
3. Using REML estimation, we tested the best model identified in stage 4 for the inclusion of random pairwise interaction effects (Brophy et al., 2017) to determine if there was remaining variability due to pairwise interactions terms.
4. In years 2 and 3, we used delta AIC to select between the best planted and realized models.

These steps follow best practice for mixed model analyses, *i.e.* to compare fixed effects using ML and to compare and estimate variance structures using REML (Littell et al., 2006).

*Additional Results*

Here we provide additional details on the fitted model for each case (year 1 planted, year 2 planted, year 2 realized, year 3 planted, and year 3 realized) and provide a table summarizing the results of the model fitting process (Table S1.1).

*Year 1 planted proportions*

The best year 1 model was M2, the average pairwise interaction model, where the single diversity effect coefficient (δ) was allowed to interact with the planted proportion of *H. maximiliani* (HM; *P_11_*):

$$y=\sum_{i=1}^{S} \beta_{i}P_{i}+\alpha_{b}+\delta_{1}\sum_{\begin{aligned} i,j=1 \\ i<j \end{aligned}}^{S} P_{i}P_{j}+\delta_{2}\sum_{\begin{aligned} i,j=1 \\ i<j \end{aligned}}^{S} P_{i}P_{j}*P_{11}+\varepsilon$$

where *P_i_* is the planted proportion of species *i* for *i*=1,…,S=16, *β_i_* is the expected monoculture performance of species *i*, *α_b_* is the effect of block *b*, *b* = 1,…,5, and *δ* and *δ’* are the diversity effect coefficients. ε is the error term, assumed independent and normally distributed with four unique variance parameters: one for HM monocultures ($\sigma_{1}^{2}$), one for all other monocultures ($\sigma_{2}^{2}$), one for mixtures with HM ($\sigma_{3}^{2}$), and one for mixtures without HM ($\sigma_{4}^{2}$). *P_11_* is the planted proportion of species HM (species 11). This flexible variance structure accommodated the higher within species variation in HM relative to that of the remaining species in our pool (Fig. 2a in main text).

*Year 2, planted proportions*

The best year 2 planted proportions model was M3, the additive species-specific interactions model, where the species-specific diversity effect terms interacted with the spatial pattern treatment (*Sp*) and the planted proportion of HM (*P_11_*):

$$y=\sum_{i=1}^{S} \beta_{i}P_{i}+\alpha_{b}+\sum_{\begin{aligned} i,j=1 \\ i<j \end{aligned}}^{S} (\lambda_{i}+\lambda_{j})P_{i}P_{j}+\sum_{\begin{aligned} i,j=1 \\ i<j \end{aligned}}^{S} (\lambda_{i}^{'}+\lambda_{j}^{'})P_{i}P_{j}*Sp+\sum_{\begin{aligned} i,j=1 \\ i<j \end{aligned}}^{S} (\lambda_{i}^{''}+\lambda_{j}^{''})P_{i}P_{j}*P_{11}+ \varepsilon$$

where *P_i_* is the planted proportion of species *i* for *i*=1,…,S=16, *Sp* is a dummy variable coded 0 for all monocultures, 0 for ‘dispersed’ plots, and 1 for ‘aggregated’ plots. ε is the error term, assumed independent and normally distributed with four unique variance parameters: one for HM monocultures ($\sigma_{1}^{2}$), one for all other monocultures ($\sigma_{2}^{2}$), one for mixtures with HM ($\sigma_{3}^{2}$), and one for mixtures without HM ($\sigma_{4}^{2}$).

*Year 2, realized proportions*

The best year 2 realized proportions model was also M3, the additive species-specific model, where the species-specific diversity effect terms interacted with the spatial pattern treatment (*Sp*) and the proportion of HM (*P_11_*):

$$y=\sum_{i=1}^{S} \beta_{i}P_{i}+\alpha_{b}+\sum_{\begin{aligned} i,j=1 \\ i<j \end{aligned}}^{S} (\lambda_{i}+\lambda_{j})P_{i}P_{j}+\sum_{\begin{aligned} i,j=1 \\ i<j \end{aligned}}^{S} (\lambda_{i}^{'}+\lambda_{j}^{'})P_{i}P_{j}*Sp+\sum_{\begin{aligned} i,j=1 \\ i<j \end{aligned}}^{S} (\lambda_{i}^{''}+\lambda_{j}^{''})P_{i}P_{j}*P_{11}+ \varepsilon$$

where *P_i_* is the realized proportion of species *i* in year 1 for *i*=1,…,S=16, ε is the error term, assumed independent and normally distributed with four unique variance parameters: one for HM monocultures ($\sigma_{1}^{2}$), one for all other monocultures ($\sigma_{2}^{2}$), one for mixtures with HM ($\sigma_{3}^{2}$), and one for mixtures without HM ($\sigma_{4}^{2}$).

*Year 3, planted proportions*

The best year 3 planted proportions model was again M3, the additive species-specific model, where species specific diversity effect terms interacted with the proportion of HM and the spatial pattern treatment (*Sp*):

$$y=\sum_{i=1}^{S} \beta_{i}P_{i}+\alpha_{b}+ \sum_{\begin{aligned} i,j=1 \\ i<j \end{aligned}}^{S} (\lambda_{i}+\lambda_{j})P_{i}P_{j}+ \sum_{\begin{aligned} i,j=1 \\ i<j \end{aligned}}^{S} (\lambda_{i}^{'}+\lambda_{j}^{'})P_{i}P_{j}*Sp+ \sum_{\begin{aligned} i,j=1 \\ i<j \end{aligned}}^{S} (\lambda_{i}^{''}+\lambda_{j}^{''})P_{i}P_{j}*P_{11}+\sum_{\begin{aligned} i,j=1 \\ i<j \end{aligned}}^{S} d_{ij}P_{i}P_{j}+\varepsilon$$

where *P_i_* is the sown proportion of species *i* for *i*=1,…,S=16. The errors are assumed to be independent and normally distributed, with a unique variance for each monoculture ($\sigma_{1}^{2}{,\ldots, \sigma}_{16}^{2}$) and a unique variance for all mixtures ($\sigma_{17}^{2}$). This model additionally included random pairwise interaction terms (*d_ij_*) assumed to be independent and normally distributed with a constant variance ($\sigma_{18}^{2}$).

*Year 3, realized proportions*

The best year 3 realized proportions model was M2, the average pairwise interaction model, with a constant diversity effect coefficient:

$$y=\sum_{i=1}^{s} \beta_{i}P_{i}+\alpha_{b}+ \delta\sum_{\begin{aligned} i,j=1 \\ i<j \end{aligned}}^{s} P_{i}P_{j}+ \varepsilon$$

where *P_i_* is the realized proportion of species *i* in year 2, for *i*=1,…,S=16. The errors are assumed to be independent and normally distributed, with a unique variance for each monoculture ($\sigma_{1}^{2}{,\ldots, \sigma}_{16}^{2}$) and a unique variance for all mixtures ($\sigma_{17}^{2}$).

**Appendix S1 References**

Brophy, C., Á. Dooley, L. Kirwan, J.A. Finn, J. McDonnell, et al. 2017a. Biodiversity and ecosystem function: Making sense of numerous species interactions in multi-species communities. Ecology 98(7): 1771–1778. doi: 10.1002/ecy.1872.

Littell, R.C., Milliken, G.A., Stroup, W.W., Wolfinger, R.D. and Oliver, S. 2006. *SAS for mixed models*. SAS publishing.

**Table S1.1.** Summary of the model fitting and selection procedure used to identify best-fit models for plot biomass production for each case (year 1 planted, year 2 planted, year 2 realized, year 3 planted, and year 3 realized). Model selection followed a six-step process (outlined earlier in appendix) and results are numbered accordingly here. The models compared were M1: identity model, M2: average pairwise interaction model, M3: additive species model and M4: functional group model. P-values provided in step 3 are from likelihood ratio tests (LRTs). The difference in AIC from the sown and realized analyses are shown with value 0 for the best of the two within each of year 2 and 3. The best model in each year is highlighted by a box.

|  |  |  |  | |  |  |  | |  |
| --- | --- | --- | --- | --- | --- | --- | --- | --- | --- |
|  | Year 1 |  | Year 2 | | |  | Year 3 | | |
|  | Planted |  | Planted | Realized | |  | Planted | Realized | |
| 1. Best model (assuming constant error variance)? | M3 |  | M2 | M1 | |  | M3 | M1 | |
| 2. Constant error variance assumption violated? (# variance parameters required) | Yes (4) |  | Yes (4) | Yes (4) | |  | Yes (17) | Yes (17) | |
| 3. Model selection, with flexible error structure: LRT p-value |  |  |  |  | |  |  |  | |
| M1 vs. M2 | 0.003 |  | <0.001 | 0.083 | |  | <0.001 | 0.008 | |
| M2 vs. M3 | 0.086 |  | 0.020 | 0.046 | |  | <0.001 | 0.143 | |
| M2 vs. M4 | 0.964 |  | 0.789 | 0.978 | |  | 0.883 | 0.883 | |
| Best model (with flexible error structure)? | M2 |  | M3 | M3 | |  | M3 | M2 | |
| 4. Additional interactions that were needed | (DE)* P_11_ |  | (DE)*P_11_ (DE)*Sp | (DE)* P_11_ (DE)*Sp | |  | (DE)* P_11_ (DE)*Sp |  | |
| 5. Random pairwise interactions needed? | No |  | No | No | |  | Yes | No | |
| 6. △ AIC (within year; 0 for best model, >0 difference from best model) |  |  | 0 | 16.5 | |  | 0 | 16.5 | |

**Appendix Figure Legends**

**Figure S1.1.** Studentized residuals versus predicted biomass values assuming constant error variance (a, c, e, g, i) and the more complex error structure selected (b, d, f, h, j) in each year and under each species proportion scenario.
