# Supplementary figures and images for: Helianthus maximiliani and species fine‐scale spatial pattern affect diversity interactions in reconstructed tallgrass prairies
Source: Ecol Evol. 2019 Oct 9;9(21):12171–81. doi: 10.1002/ece3.5696 (PMC6854329; doi:10.1002/ece3.5696)

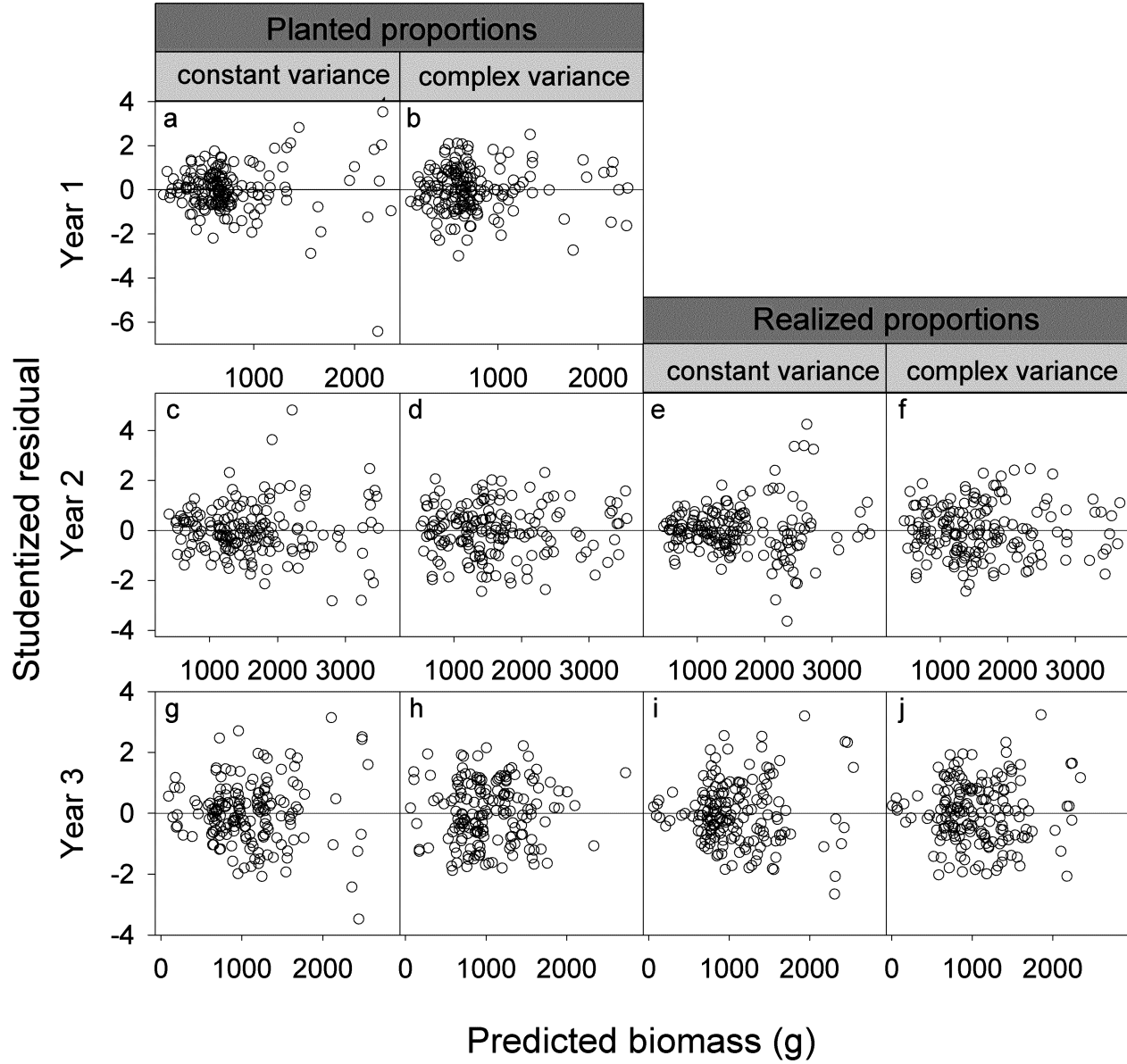

Supplement: Supplementary file 2 [file ECE3-9-12171-s002.PDF]
